# Interlaminar stabilization and decompression for the treatment of bilateral juxtafacet cysts: Case report and literature review

**DOI:** 10.1016/j.ijscr.2019.03.047

**Published:** 2019-03-30

**Authors:** Iahn Cajigas, Alberto Varon, Howard B. Levene

**Affiliations:** a Department of Neurological Surgery, University of Miami Miller School of Medicine, Miami, FL, USA; b Facultad de Ciencias de la Salud, Universidad Anáhuac México North Campus, Naucalpan de Juárez, Estado de México, Mexico

**Keywords:** JFC, juxtafacet cyst, ILD, interlaminar stabilization device, Spinal stenosis, Synovial cysts, Laminectomy, Prostheses, Coflex, Interlaminar stabilization device

## Abstract

**Introduction:**

Lumbar juxtafacet cysts (JFCs) are a common cause of lumbar radiculopathy which tend to occur in areas of increased facet mobility. While resection alone is a possible treatment, recent publications suggest that laminectomy alone for JFCs may not yield as favorable an outcome as laminotomies reinforced with posterior dynamic hardware. The Coflex^®^ is a novel interlaminar stabilization device that has been shown to achieve comparable results to rigid fusion in the management of lumbar stenosis in patients with no more than grade one anterolisthesis, and superior performance compared to laminectomy alone when a combined outcome score was used. We describe the combined use of dynamic posterior element fusion with primary cyst resection in the management of bilateral JFCs.

**Presentation of case:**

A 71-year-old man who developed a progressive left L4 radiculopathy along with new urinary incontinence was found to have bilateral L3/4 JFCs causing significant lumbar stenosis and neurogenic claudication. After treatment with primary cyst resection and interlaminar stabilization, the patient experienced complete symptom resolution and was discharged to inpatient-rehabilitation on post-operative day 1.

**Discussion:**

While current recommendations for the management of juxtafacet cysts causing progressive neurologic symptoms include surgical cyst removal and lumbar decompression with or without fusion, the role of dynamic interlaminar stabilization has not been explored.

**Conclusion:**

Direct decompression followed by interlaminar stabilization may represent an alternative for patients to simultaneously benefit from a decompression of their juxtafacet cysts while affording posterior element reconstruction.

## Highlights

• Lumbar juxtafacet cysts are typically treated by resection alone or resection combined with posterior instrumentation.
• Resection with instrumentation is associated with a lower rate of recurrence but also with increased cost and morbidity.
• We present a case of bilateral juxtafacet cysts causing neurogenic claudication treated with decompression and interlaminar stabilization.
• Complete symptom resolution was sustained at one-year follow-up.
• Decompression followed by interlaminar stabilization may be a reasonable alternative for some patients.

## 1 Introduction

Articular cysts were first described by Aberk von Gruker during an autopsy procedure in 1880 [1] and in 1885 Baker described pathologic cysts adjacent to articulations [2]. The term “juxtafacet cyst” (JFC) was first utilized by Kao et al. in 1974 to properly name both synovial and ganglion cysts adjacent to the periarticular connective tissue of the facet joints along with cysts that arise from or into the ligamentum flavum [3].

The prevalence of lumbar JFC has been reported at approximately 0.6%, with appearance at a mean age of 64 years and with a slight female predominance (51.2% vs 48.8%) [4]. While juxtafacet cysts may be asymptomatic and found incidentally [5], the most common presenting symptoms are radiculopathy (69.6%), back pain (48.3%) and neurogenic claudication (28.2%). Sensory deficits may be present in up to 34.6% of patients while motor deficits can be found in 20.8% with a mean duration of symptoms before surgery of 7.5 months [6].

Juxtafacet cysts may occur in the cervical (<5%) or thoracic (<5%) regions, however they are most frequent in the lumbo-sacral region (>90%). [7] The majority of the juxtafacet cysts are found at L4-L5 and their presence at this level is thought to be suggestive of their pathogenesis since this is considered the most mobile lumbar level and is also the site of maximum instability [8].

The following case is presented according to the SCARE criteria consensus guidelines for surgical case reports [9].

## 2 Case report

**Fig. 1:**
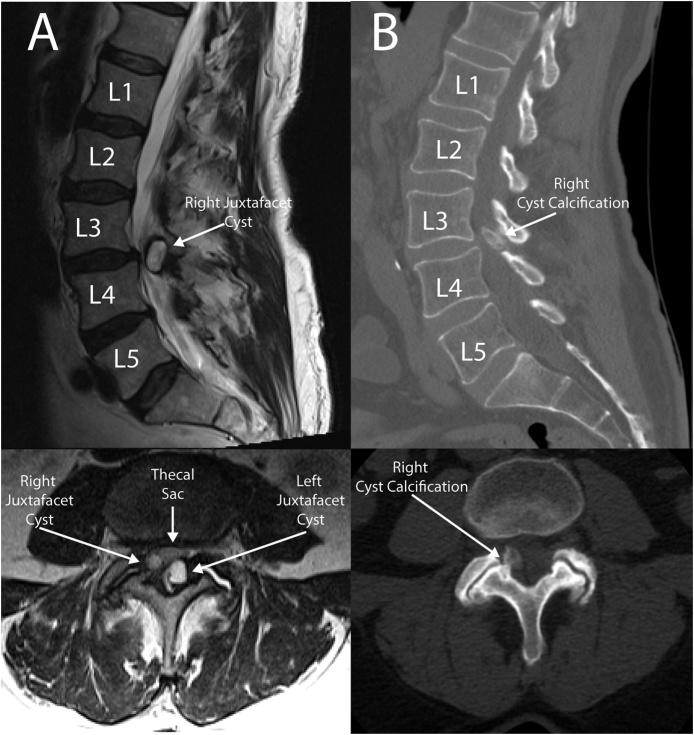
A) Pre-operative MRI (sagittal-top, axial-bottom) demonstrates bilateral juxtafacet cysts (left > right). B) Pre-operative CT (sagittal-top, axial-bottom) shows calcification of the smaller right-sided cyst.

A 71-year-old gentleman with a history of degenerative left hip osteoarthritis status post left hip arthroplasty 2 months prior presented for evaluation due to several weeks of a worsening back and left leg pain, and new urinary retention with overflow incontinence. Physical exam was notable for pain-limited weakness of the left leg in all muscle groups (4+/5 strength on manual muscle testing) and decreased sensation to light touch from the anterolateral thigh down to the dorsum of the left foot and toe in an L4 dermatomal distribution. As shown in Fig. 1, lumbar MRI demonstrated severe thecal sac stenosis at L3-L4 secondary to bilateral, left greater than right juxtafacet cysts causing compression of the cauda equina nerve roots. A non-contrast computed tomographic (CT) scan showed severe L3-L4 stenosis and lumbar flexion/extension films showed no instability. After discussion of various treatment options, the patient agreed to undergo direct removal of the JFCs with placement of the Coflex^®^ device for dynamic stabilization to avoid a single-level instrumented fusion while attempting to minimize the risk of cyst recurrence. Surgery was performed three days after initial clinic presentation.

**Fig. 2:**
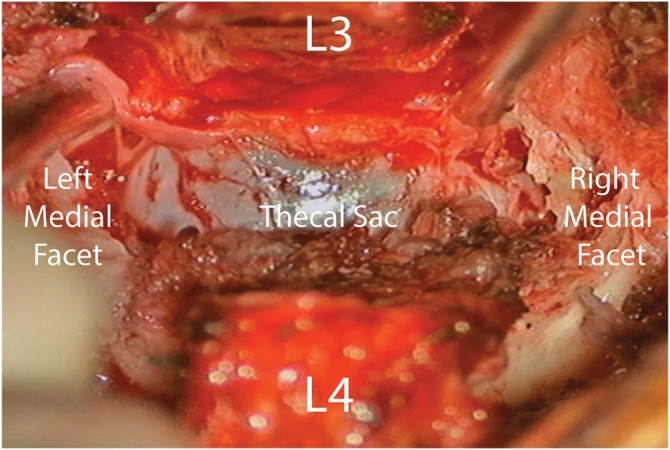
Intraoperative view prior to ILD placement at the completion of partial L3 and L4 laminectomies, bilateral cyst resection, and mesial facetectomies showing a decompressed thecal sac.

**Fig. 3:**
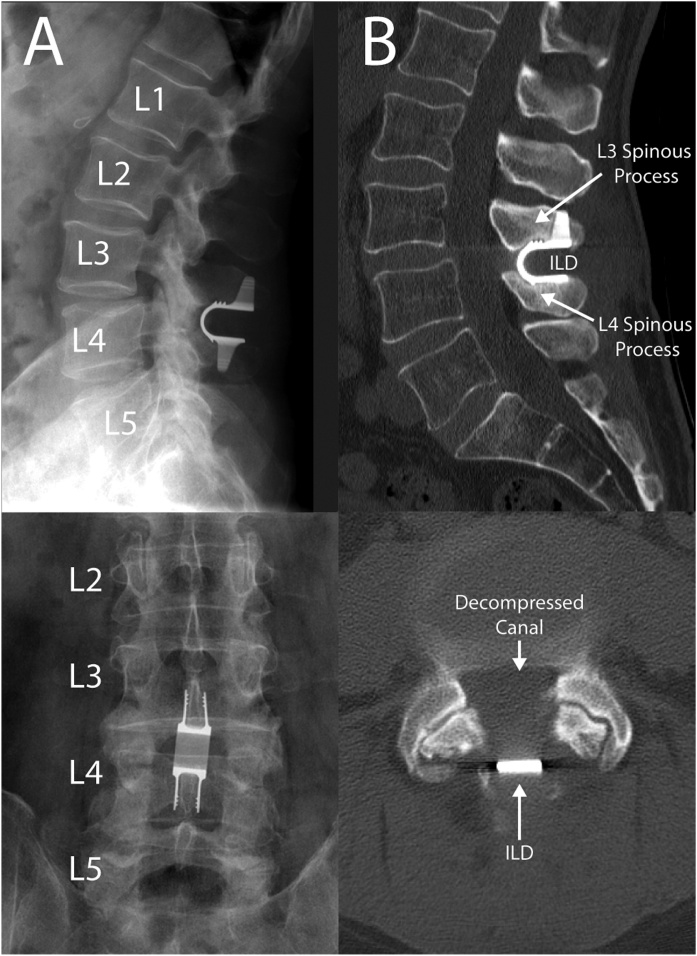
A) Post-operative X-Rays (lateral-top, antero-posterior-bottom) showing ILD at L3-4 obtained on post-operative day 1. B) 6-month post-operative CT (sagittal-top, axial-bottom) shows hardware in place with no evidence of cyst recurrence.

Decompression of the thecal sac at L3-4 was accomplished via partial inferior L3 and superior L4 laminectomies and mesial facetectomies via a standard midline approach in the usual fashion (Fig. 2). The juxtafacet cysts were seen bilaterally and completely excised using microsurgical techniques by the senior author. After decompression, a Coflex^®^ (Paradigm Spine, LCC, New York, NY) implant was placed between the remaining L3 and L4 lamina and spinous processes and the tissues were closed in the usual fashion. Total estimated blood loss was approximately 35cc. Juxtafacet cysts were confirmed on pathology. Postoperatively, the patient had immediate improvement in his left leg pain and no complications. The patient was transferred to inpatient rehabilitation on post-operative day 1. At 1-year follow-up the patient continues with complete resolution of his left leg pain and urinary symptoms. Fig. 3 demonstrates post-operative standing films and 6-month post-operative CT.

## 3 Discussion

### 3.1 Current Management Recommendations

Current management guidelines are still a matter of debate and there have been several reports of spontaneous regression of synovial cysts with one of them resolving suddenly before surgery. [10] Evidence of spontaneous regression supports the idea that conservative management may be sufficient for treating juxtafacet cysts in the absence of progressive neurologic deficits. Spontaneous regression did not occur in this patient as the cysts were clearly visualized at the time of surgery and confirmed on pathology. Non-operative options include expectant therapy, bed rest, NSAIDs, physiotherapy, orthopedic corsets, chiropractic care, CT-guided needle aspiration and intra-articular corticosteroids injections. Typically, non-operative management strategies are pursued in the absence of significant neurologic deficits. However, non-operative management is generally not effective, giving only short-term or no relief at all [7,11]. Khan & Girardi suggested that non-operative options should be tried for a period of 6 months after which surgical treatment needs to be considered [8].

Current recommendations for the management of juxtafacet cysts causing progressive neurologic symptoms include surgical cyst removal and lumbar decompression with or without fusion [7,12]. While fusion may be recommended in patients with JFCs that have preoperative symptoms of spinal instability, the role of fusion is still debated [12]. A retrospective study by Xu et al. reported a higher risk of recurrent back pain in patients that received laminectomy alone versus laminectomy and instrumented fusion (p = .018) [6]. Khan et al. showed that patients having concomitant fusion tended to have better patient reported outcomes compared to decompression alone (80% vs 70%) [8].

While spinal fusion is associated with a lower rate of cyst recurrence and improved pain control, it is associated with increased perioperative morbidity, length of hospital stay, and intraoperative blood loss compared with decompression alone [6,13]. In a systematic review by Bydon et al. of 966 patients in which 84% (n = 811) received surgical resection alone and 16% (n = 155) received resection and fusion, same-level juxtafacet cysts recurrence occurred in 1.8% patients in the resection alone group compared to no (0%) recurrence in the resection and fusion group. Same-site synovial cyst recurrence has never been reported in any patients receiving concomitant spinal fusion [6].

Fusion surgery accelerates the degeneration of adjacent segments by eliminating the fixed segment motion and by increasing the motion at unfused adjacent segments a point that may be of significant importance for patients with bilateral cysts [14]. There have been some proposed alternatives to rigid fusion. Interlaminar stabilization devices (ILD), such as the Coflex®, are placed between adjacent lamina and spinous processes following decompression surgery (bilateral laminotomies, foraminotomies, mesial facetectomies) and have been shown to preserve both flexion and extension while stabilizing adjacent vertebra [15]. The reduced stiffness of the ILD compared to rigid fusion permits more physiological load transmission.

While the use of ILDs has not been previously reported for the treatment of bilateral juxtafacet cysts, numerous studies have demonstrated that ILDs have utility in the management of lumbar stenosis. Most recently, Schmidt et al. conducted a clinical trial comparing decompression alone (DA) vs decompression + interlaminar stabilization (D + ILS) for the management of lumbar stenosis and demonstrated that while there was no significant difference in individual patient reported outcomes at 24 month follow-up, the composite clinical success was significantly better in the D + ILS group than the DA group [16]. Overall, the D + ILS cohort did better than DA in various measures, e.g. there was more risk of secondary intervention on the DA cohort (p = 0.055); 228% more lumbar injections required (p = 0.0065); higher rate of narcotic use at every time point post surgically (16.7% for D + ILS vs 23% for DA at 24 months). In other words, there is a clinical benefit to reconstructing the posterior elements after a decompression.

ILDs may serve as a good alternative to rigid fusion. Bae et al. compared patients with lumbar spinal stenosis undergoing posterolateral spinal fusion after decompression surgery and those receiving interlaminar stabilization procedure (ISP) with Coflex^®^ there was no significant difference in the number of reoperations needed [17]. Kumar et al. demonstrated that patients who underwent placement of an ILD for lumbar stenosis, had significant functional improvement as measured in their ODI and Visual Analogue Scale (VAS) back and leg pain scores compared to those receiving decompression alone [18]. In a meta-analysis of 7 cohorts, Li et al. showed that decompression + Coflex^®^ interlaminar stabilization was not inferior in terms of functional clinical outcome (ODI and VAS pain scores) and that it was associated with significantly shorter length of stay and blood loss compared to decompression with concomitant fusion [19]. Major device-related complications have only been reported in two studies which demonstrated no significant difference in decompression + Coflex^®^ compared to decompression with concomitant fusion [19]. In the case presented here, an ILD was selected as an alternative to rigid fusion after bilateral cyst decompression to minimize the risk of cyst recurrence and the morbidity of rigid fusion.

## 4 Conclusion

We present a novel surgical option for the treatment of bilateral JFCs causing lumbar stenosis with neurogenic claudication where rigid fusion is avoided, and dynamic fusion is employed to reconstruct the posterior elements. This surgical option has the benefits of minimal blood loss, decreased length of surgery, and faster patient recovery, and avoidance of rigid fusion. While this condition is uncommon, our approach presents a viable treatment alternative that should be considered for patients with bilateral juxtafacet cysts, no instability on preoperative lumbar x-rays and a maximum of grade one anterolisthesis.

## Conflict of interest

None.

## Sources of funding

None.

## Ethical approval

The patient provided verbal consent for publication of this case report. Institutional IRB approval not required.

## Consent

Informed consent statement has been added to the manuscript. Written informed consent was obtained from the patient for publication of this case report and accompanying images. A copy of the written consent is available for review by the Editor-in-Chief of this journal on request.

## Author contribution

**Iahn Cajigas:** Conceptualization, Data curation, Investigation, Writing-Original draft preparation, Writing- Review and Editing.

**Alberto Varon:** Data curation, Investigation, Writing-Original draft preparation, Writing- Review and Editing.

**Howard B. Levene:** Conceptualization, Writing- Review and Editing, Supervision.

## Registration of research studies

This study does not represent a prospective research study but rather a single off label use of an FDA approved device.

## Guarantor

Howard Levene (senior author).

## Provenance and peer review

Not commissioned, externally peer-reviewed.
